# Overall Survival of Stage III Colon Cancer with Only One Lymph Node Metastasis Is Independently Predicted by Preoperative Carcinoembryonic Antigen Level and Lymph Node Sampling Status

**DOI:** 10.1371/journal.pone.0137053

**Published:** 2015-09-18

**Authors:** Been-Ren Lin, Yu-Lin Lin, Hong-Shiee Lai, Po-Huang Lee, King-Jen Chang, Jin-Tung Liang

**Affiliations:** 1 Division of Colorectal Surgery, Department of Surgery, National Taiwan University Hospital and College of Medicine, No. 7, Chung-Shan South Road, Taipei, Taiwan, ROC; 2 Department of Oncology, National Taiwan University Hospital and College of Medicine, No. 7, Chung-Shan South Road, Taipei, Taiwan, ROC; University of Florida, UNITED STATES

## Abstract

**Background:**

This study identified predictors of favorable overall survival (OS) for stage III colon cancer patients who had only one lymph node (LN) metastasis (N1a).

**Methods:**

Variables, including preoperative carcinoembryonic antigen (CEA) level, LN sampling status, and the choices of postoperative adjuvant chemotherapy, were recorded. Prognostic significance was determined using the log-rank test and multivariate Cox regression analysis.

**Results:**

The median 42-month follow-up period included 363 eligible patients. Among them, 230 (63.3%) received only 5-flurouracil (5-FU) adjuvant chemotherapy; 76 (20.9%) underwent oxaliplatin-based regimens; and 57 (15.7%) chose surgery alone. The 5-year survival rate of these evaluated patients was 75%, 63%, and 77%, respectively (P = 0.823). Multivariate analysis revealed that normal preoperative CEA level (≦5 ng/mL) and adequate LN sampling (LN ≧ 12) were significant predictors for higher 5-year OS (P < 0.001; P = 0.007, respectively). However, the use of postoperative adjuvant chemotherapy in these N1a colon cancer patients did not significantly affect their 5-year OS.

**Conclusions:**

A preoperative CEA level of less than or equal to 5 ng/mL, and curative surgery with an adequate lymphadenectomy determined a favorable OS outcome in stage III colon cancer with only one LN metastasis.

## Introduction

Colon cancer is one of the most common malignancies and the leading cause of cancer-related death in Europe and the United States [1]. Colon cancer is also the third leading cause of cancer death in Taiwan, and its incidence is rapidly increasing [2]. Patient prognosis primarily relies on the tumor stage at diagnosis. Although more than two-thirds of all colon cancer patients undergo radical surgery, 30% to 50% of patients with stage II or stage III tumors inevitably experience tumor relapse manifesting as locoregional recurrence, distant metastasis, or metachronous colorectal lesions within 5 years of follow up [3]. Therefore, postoperative adjuvant chemotherapy with 5-flurouracil (5-FU) plus leucovorin has been widely recommended as the standard treatment for stage III colon cancer since the early 1990s [4], and has resulted in a 30% decrease in the relapse rates compared with surgery alone. The recent addition of oxaliplatin to the 5-FU/leucovorin regimens has further improved the outcome of stage III colorectal cancer (CRC) patients, and these approaches are becoming accepted as a new standard of care [5]. However, in a report of a population-based sample of Medicare enrollees diagnosed with stage III colon cancer, only 55% of these patients received postoperative adjuvant chemotherapy [6]. This actual medical practice revealed that not every stage III CRC patient receives postoperative adjuvant chemotherapy.

The tumor-node-metastasis (TNM) system developed by the American Joint Committee on Cancer (AJCC) is an internationally recognized method for evaluating stages of colon cancer. The sixth edition of the AJCC’s system subdivided stage III disease into IIIA (T1-2N1), IIIB (T3-4N1), and IIIC (any TN2),[7] and this version of staging was validated based on data from the Surveillance, Epidemiology, and End Results (SEER) program [3]. However, this national-based population survival result revealed a conflicting finding of longer overall survival (OS) among patients with stage IIIA disease than those with stage IIB (T4N0) disease. Therefore, the AJCC revised its sixth edition of colon cancer staging to the seventh edition in 2009 [8]. Aside from certain improvements, the primary substaging principle in the seventh edition remains unchanged. One change subdivided the N1 into N1a (one positive lymph node) and N1b (two or three positive lymph nodes). The large SEER colon cancer analysis further validated the merits of the seventh edition, demonstrating that patients with N1a have a 5% to 13% enhanced 5-year OS rate than those with N1b in the same T-category [9]. We noted that patients with T1-2N1a (stage IIIA) have a similar 5-year OS compared with those with T2N0 (stage I) or T3N0 (stage IIA) stages [73.7% (T1-2N1a), 74.3% (T2N0) and 66.7% (T3N0), respectively].

Therefore, stage III colon cancer patients with only one lymph node (LN) metastasis (N1a) might have an equivalent 5-year OS compared with certain colon cancer patients diagnosed with stage I (T2N0) or stage IIA (T3N0). In this select group of patients, scant published data have described the factors that affect tumor recurrence or OS. Therefore, we attempted to identify the favorable prognostic factors by comparing patients who did and did not receive adjuvant chemotherapy. Defining the percentage of these stage III N1a patients who might not require postoperative adjuvant chemotherapy is crucial for preventing patients from experiencing chemotherapy toxicity and side effects.

## Methods

Stage III colon cancer patients were retrospectively identified from lists obtained from the Medical Information Management Office and the Cancer Registry Office of National Taiwan University Hospital (NTUH) from December 2004 to July 2010, which contain the recorded and analyzed clinical and pathological data of eligible patients. This study was approved by the Institutional Review Board of NTUH. Patients provided written informed consent to participate in this study, and the Ethics Committee of NTUH approved the consent procedure.

All resections were completed with curative intent, which included the primary colonic lesions, removed adjacent organs, and all resected LNs. Because of the complexity of rectal cancer treatment, including neoadjuvant radio-chemotherapy and post-operative adjuvant chemotherapy, patients with rectal cancer were excluded in our study. All surgery was performed by attending surgeons subspecialized in managing colorectal cancer. Emergency operations for colon obstruction or perforation, and resection for recurrent diseases were excluded in this analysis. Diagnosis of colon cancer was established by reviewing the morphology of cancer cells and immunohistochemistry (CK20 or CDX2) of pathological specimens by two independent pathologists. The clinical decision of postoperative chemotherapy was based on a discussion with patients about the advantages and disadvantages of receiving adjuvant chemotherapy, the potential complications and side effects after treatments, the existence of high-risk factors that may lead to recurrence and compromise patients’ outcome, and finally, their preferences. Over the study period, two options of adjuvant chemotherapy were available: (1) infusional 5FU/leucovorin alone, and (2) oxaliplatin/5FU/leucovorin. Infusional 5FU/leucovorin consisted of 5-FU 1500 mg/m^2^ plus leucovorin 75 mg/m^2^, intravenous infusion for 20 hours for 2 days, repeated every 2 weeks for a total of 6 months. The oxaliplatin-based chemotherapy consisted of leucovorin 400 mg/m^2^ infusion for 2 hours before 5-FU followed by 5-FU 400 mg/m^2^ bolus on Day 1, and 5FU 2400 mg/m^2^ infusion over 46 hours; oxaliplatin 85 mg/m^2^ infusion on Day 1, repeated every 2 weeks for a total of 6 months.

All patients received regular follow-up consisting of periodic physical examinations, blood chemistry panels (such as complete blood cell count and liver function tests), carcinoembryonic antigen (CEA) level, colonic endoscopy, and abdominal ultrasonography and radiographs of the thorax. Computed tomography (CT) or magnetic resonance imaging (MRI) was also performed in cases where tumor recurrence was suspected.

## Statistical Analysis

We used the chi-square test for comparing categorical variables. Disease-free survival (DFS) was measured from the date of the primary colonic surgery to the date of recurrence. The OS time was calculated from the date of surgery to the time of the last visit or death. Follow-up was updated in January 2014 in the current study. Censor was recorded on the date locked for patients who were free of recurrence, who were alive, or who were lost to follow-up. Therefore, patients with incomplete follow-up or those lost to follow-up were still included in this study. We estimated each variable factor of survival rate by using the Kaplan–Meier method. The significance of differences between subgroups was calculated using the log-rank test. Multivariate Cox regression analysis with stepwise selection was used to search for independent prognostic factors associated with survival. A P value < 0.05 was considered statistically significant. All tests were two-sided. These analyses were performed using SPSS Version 16.0 for Windows and the patient data used in this study can be found in S1 Dataset.

## Results

During the median 42-month follow-up period (range 18–103 months), 363 stage III colon cancer patients exhibiting only one LN metastasis (N1a) were treated and followed at NTUH. Among them, 230 (63.3%) received 5-FU alone postoperative adjuvant chemotherapy; 76 (20.9%) underwent oxaliplatin-based regimens, and 57 (15.7%) underwent surgery alone (no chemotherapy). The reasons for not receiving chemotherapy included patient comorbidities (12 cases), patient preference (26 cases), favorable pathology as deemed by surgeons (5 cases), and no detailed medical documents (14 cases). Descriptive clinical features and tumor characteristics of patients who did and did not receive adjuvant chemotherapy are detailed in Table 1. Except for age, which was a crucial determinant for choosing postoperative treatment, the remaining critical prognostic factors, including sex, performance status, location of the primary tumor, T stage, differentiation, preoperative CEA level, invasion, and the number of LN samplings were nonsignificantly different among these three groups. We observed that the age of the patients in the group of oxaliplatin-based regimens was significantly younger than those in the 5FU alone group or the no-chemotherapy group because the efficacy and side effects of oxaliplatin-based regimens might be stronger than those of the 5FU alone regimens or no chemotherapy.

**Table 1:** Clinicopathological features in stage III CRC patients with only one lymph node metastasis according the different treatment regimens.

	5FU alone regimens (n = 230)	Oxaliplatin-based regimens (n = 76)	No Chemotherapy (n = 57)	*P* value
Age	64.84 ± 12.506	58.66 ± 12.021	66.77 ± 12.865	<0.001
Performance				0.174
0 ~ 1	222	76	54	
2	8	0	3	
Sex				0.960
Male	128	41	31	
Female	102	35	26	
Location				0.254
Right	81	22	16	
Left	98	28	27	
Sigmoid	51	26	14	
T stage				0.069
T1 T2	41	23	12	
T3 T4	189	53	45	
Differentiation				0.599
Well	6	1	0	
Moderate	216	74	56	
Poor	7	7	1	
CEA level				0.272
≦5	153	54	44	
>5	77	22	13	
Invasion				0.347
Positive	65	27	20	
negative	165	49	56	
LN sampling				0.128
< 12	80	17	19	
≧12	150	59	38	

**Fig 1:**
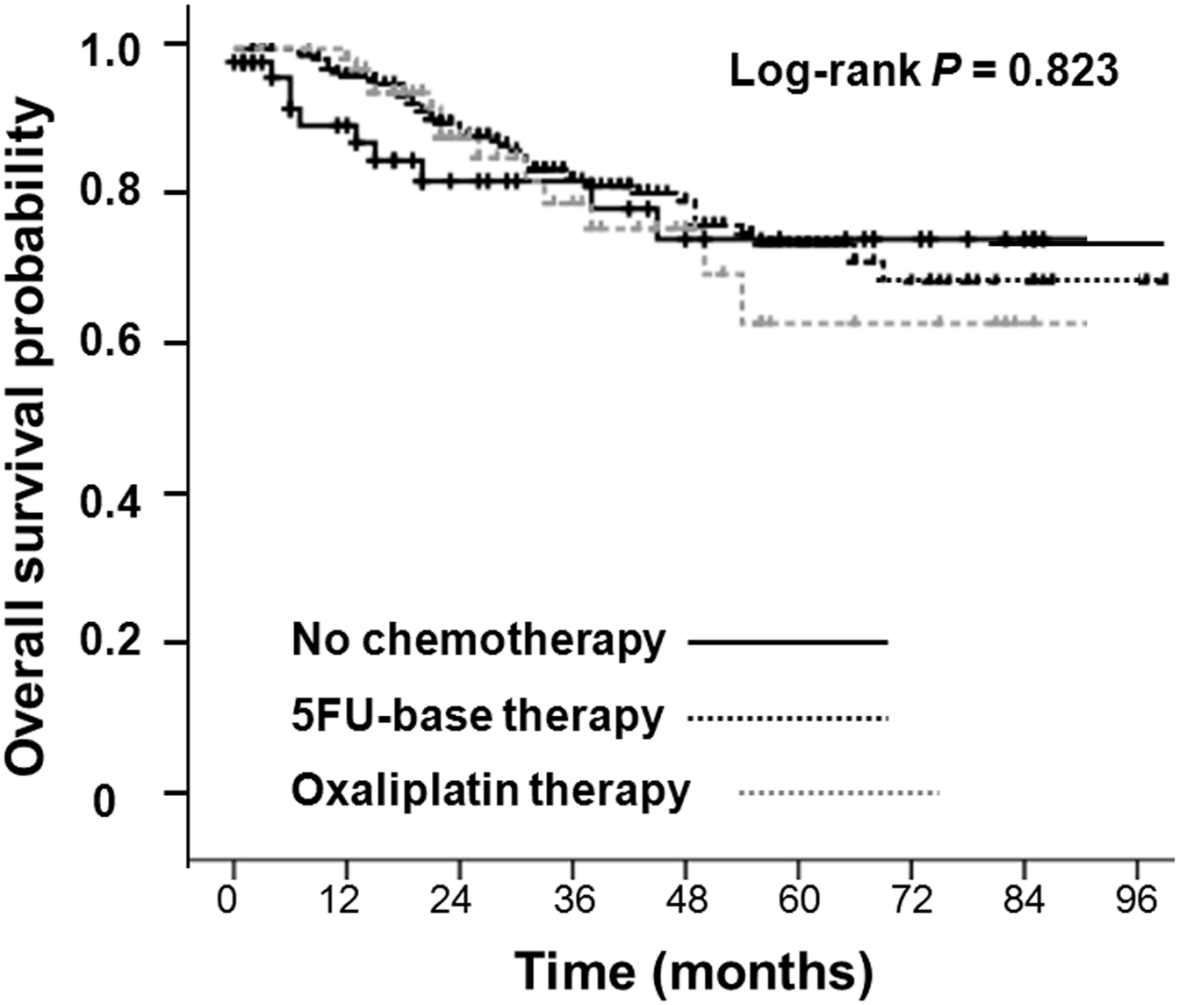
Overall survival in N1a colon cancer who had used and never-used chemotherapy regimens.

We explored potential predictive and prognostic factors that may influence patient outcomes and found that a preoperative CEA level of ≦ 5 ng/mL and an LN sampling of ≧ 12 during operation were both more accurate predictive and prognostic factors for enhanced 5-year DFS and 5-year OS (Table 2). When these stage III colon cancer patients who had only one LN metastasis (N1a) received different postoperative adjuvant chemotherapies, the 5-year OS among various groups was nonsignificantly different (Fig 1). The 5-year OS rate was 75% in the patient group who received 5FU alone regimens, 63% in the patient group who underwent oxaliplatin-based regimens, and 77% in the patient group who received only observation (P = 0.823). Furthermore adjuvant chemotherapy did not influence the 5-year DFS of these stage III patients who had only one LN metastasis (N1a) (Table 2).

**Table 2:** Univariate analysis for 5-year overall and 5-year disease-free survival in 363 patients with colon cancer and only one lymph node metastasis.

Characteristics	Patient Number	5-year survival rate	*P* value	5-year disease-free	*P* value
Age			0.061		0.835
< 75	287	65.6%		52.2%	
≧ 75	76	77.6%		61.4%	
Gender			0.201		0.042
Male	200	72.8%		56.7%	
Female	163	78.5%		68.2%	
Performance^Δ^			0.999		0.426
0~1	352	84.2%		77.1%	
2	11	78.2%		72.8%	
Location^Ψ^			0.518		0.503
Right	119	76.7%		61.4%	
Left	153	72.5%		60.8%	
Sigmoid	91	71.2%		52.3%	
Differentiation			0.782		0.938
Well	7	85.6%		72.5%	
Moderate	346	73.6%		59.1%	
Poor	9	84.7%		56.2%	
Tumor depth			0.314		0.026
T1+2	76	79.1%		72.4%	
T3+4	287	72.6%		54.7%	
Invasion^Ω^			0.573		0.218
Present	250	72.8%		58.1%	
Absent	113	73.3%		61.7%	
CEA level			**< 0.001**		**< 0.001**
≦5	251	81.2%		69.2%	
>5	112	54.6%		38.9%	
LN sampling number			**0.012**		**0.027**
≧ 12	247	78.9%		68.3%	
< 12	116	64.6%		47.5%	
Treatment			0.0692		0.150
Chemotherapy(-)	57	76.1%		78.7%	
Chemotherapy(+)	306	73.2%		57.8%	

^Δ^ Performance status: according to the definition of ECOG-WHO

^Ψ^ Location: Right: cecum to middle transverse colon, Left: middle transverse to descending colon.

^Ω^ Invasion (present): if pathological report revealed one of venous, lympho-vessel or perineural invasion

**Fig 2:**
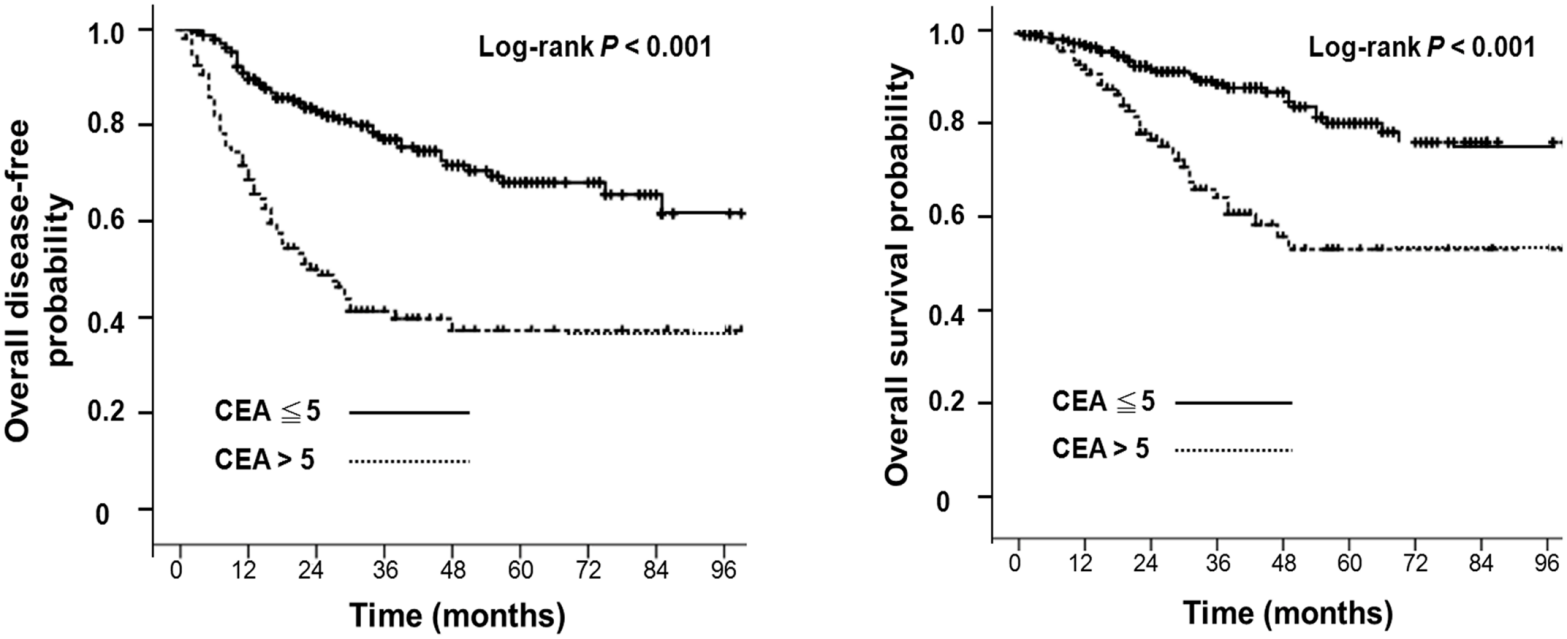
Fig 2A: Disease-free survival in N1a colon cancer patients, divided by pre-operative carinoembryonic antigen (CEA) level. Fig 2B: Overall survival in N1a colon cancer patients, divided by pre-operative carinoembryonic antigen (CEA) level.

**Fig 3:**
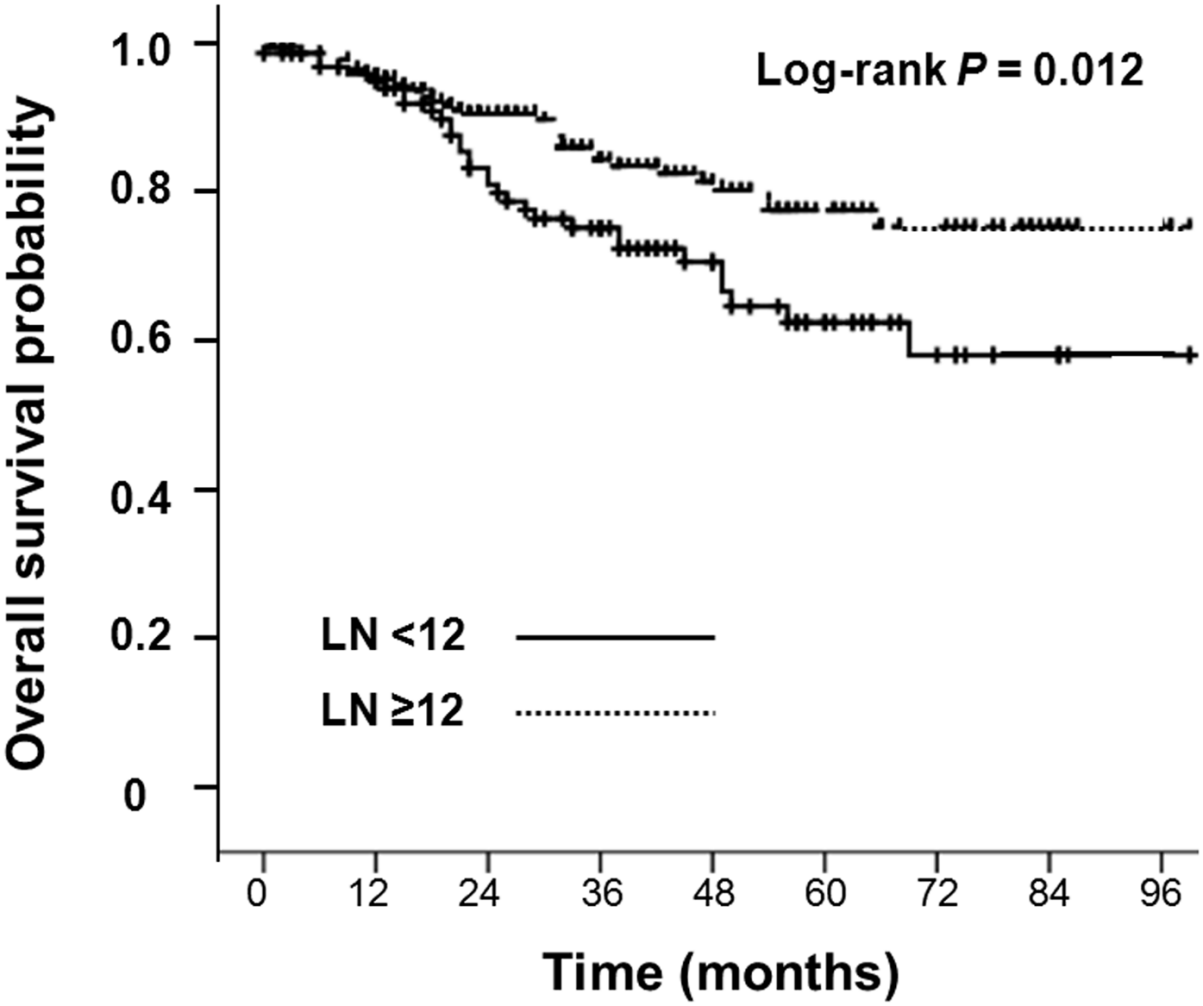
Overall survival in N1a colon cancer patients, divided by lymph node sampling status.

In multivariate analysis, the preoperative CEA level of ≦ 5 ng/mL remained independently predictive of prognostic factors for enhanced 5-year DFS and 5-year OS. The 5-year DFS rate in patients with a preoperative CEA level of ≦ 5 ng/mL and > 5 ng/mL was 69.2% and 38.9%, respectively (P < 0.001; hazard ratio [HR], 3.28; 95% confidence interval [CI], 2.24–4.81) (Fig 2A). In addition, the 5-year OS in patients with preoperative CEA level of ≦ 5 ng/mL and > 5 ng/mL was 81.2% and 54.6%, respectively (P < 0.001; HR: 3.42; 95% CI, 2.05–5.73) (Fig 2B). However, LN sampling of ≧ 12 during operation, in multivariate analysis, was another independent prognostic factor for higher 5-year OS (Table 3). The 5-year OS in patients with LN sampling ≥ 12 and < 12 was 78.9% and 64.6%, respectively (P = 0.007; HR: 2.11; 95% CI, 1.28–3.41) (Fig 3).

**Table 3:** Results of multivariate Cox regression modeling of overall survival and recurrence-free survival in N1a colon cancer.

Characteristics		Overall survival			Disease-free rate		
		Hazard ratio	95% CI	*P* value	Hazard ratio	95% CI	*P* value
Age	< 75	1.00		0.442	1.00		0.997
	≧ 75	0.98	0.96 ~ 1.02		0.99	0.97 ~ 1.02	
Gender	male	1.00		0.384	1.00		0.099
	female	0.92	0.89 ~ 1.12		0.95	0.62 ~1.54	
Performance	0~1	1.00		0.949	1.00		0.280
	2	1.07	0.14 ~ 8.07		0.57	0.21 ~ 1.58	
Location	right	1.00		0.391	1.00		0.598
	left	0.63	0.33 ~ 1.22	0.171	0.78	0.49 ~ 1.26	0.313
	sigmoid	0.78	0.43 ~ 1.43	0.418	0.86	0.55 ~ 1.35	0.515
Differentiation	well	1.00		0.349	1.00		0.719
	moderate	2.19	0.19 ~ 25.21	0.529	0.55	0.09 ~ 3.45	0.527
	poor	0.75	0.10 ~ 5.57	0.777	0.62	0.19 ~ 2.01	0.428
Tumor depth	T1+2	1.00			1.00		0.125
	T3+4	1.129	0.51 ~ 2.52	0.767	1.48	0.89 ~ 2.44	
Invasion	Present	1.08	0.63 ~ 1.89	0.765	0.94	0.62 ~ 1.43	0.783
	Absent	1.00			1.00		
CEA level	≦5	1.00			1.00		
	> 5	3.42	2.05 ~ 5.73	<0.001	3.28	2.24 ~ 4.81	<0.001
LN sampling number	≧ 12	1.00			1.00		
	< 12	2.11	1.28 ~ 3.41	0.007	1.48	0.98 ~ 2.16	0.056
Treatment of chemotherapy	(+)	1.21	0.61 ~ 2.42	0.591	0.69	0.37 ~ 1.31	0.262
	(-)	1.00			1.00		

## Discussion

In this study, we demonstrated that stage III colon cancer with only one LN metastasis might represent a unique patient population for the following reasons. (1) Stage III colon cancer patients who have only one LN metastasis, based on the seventh AJCC stage classification, belong to different substages, including stage IIIA (T1-2N1aM0), IIIB (T3-4N1aM0), or IIIC (T4aN1aM0). (2) Stage IIIA, IIIB, and IIIC patients typically manifest different DFS and OS. (3) According to the consensus [4] and randomized clinical trial result [5], postoperative adjuvant chemotherapy is recommended for all stage IIIA, IIIB, and IIIC CRC patients. However, in our analysis, the 5-year OS of stage III colon cancer patients with only one LN metastasis who did or did not receive adjuvant chemotherapy was nonsignificantly different. By contrast, our results unveiled two independent favorable prognostic factors, including a preoperative CEA level of ≦ 5 ng/mL and LN sampling ≧ 12 during operation, for enhanced 5-year OS.

Numerous reports have indicated that a low preoperative CEA level represents low risk of relapse [10,11]. In a large SEER database result (N = 17,910), the abnormal preoperative CEA level (> 5 ng/mL) was independently associated with a 60% increased risk of overall mortality (HR of death = 1.60, 95% CI = 1.46–1.76, P < 0.001). [12] However, an adequate LN harvest during surgery is also crucial for accurately defining disease stage. The suggested number of LNs to be removed during surgery for accurate staging has increased from nine in a 2002 study [13] to at least 12 in recent studies [14–19], which our multivariate analysis findings support. Furthermore, using a subgroup analysis for adequately staged (> 12 LNs) N1a patients revealed that preoperative CEA levels were the most crucial prognostic factor for overall disease-free survival (P < 0.001). Administering adjuvant chemotherapy did not significantly affect their survival. Therefore, our study indicated that a preoperative CEA level of ≦ 5 ng/mL and an adequate LN sampling (≧ 12) during surgery were independent prognostic factors for a higher 5-year OS. These two factors might define superior candidates who do not require adjuvant chemotherapy within stage III colon cancer patients with only one LN metastasis. However, our study findings must be tested only in prospective randomizing settings.

This study has several limitations. First, because the study was retrospective, concluding that adjuvant chemotherapy conferred no survival benefits in patients with one positive LN is inappropriate. Moreover, the study period included changes in the adjuvant chemotherapeutic regimens, from 5FU/leucovorin in the early era to oxaliplatin-based in the later study period. Because of the small number of patients receiving oxaliplatin, it was not possible to determine whether a more modern adjuvant chemotherapeutic regimen was associated with enhanced survival. In our study, patients in the oxaliplatin-based regimen group were younger than those in the 5FU alone group and the no-chemotherapy group. This finding reflected actual practice, that the younger the patients, the stronger the adjuvant chemotherapy regimens that are administered [6,20,21]. The fact that older patients receive fewer or weaker adjuvant chemotherapies is typically based on physicians’ clinical judgment and evidence from clinical study. For example, in a large sample size study (14,528 patients) evaluating the effect of age on the efficacy of adjuvant therapies from seven adjuvant therapy trials, the authors concluded that older patients (age > 70 years) experienced reduced benefit from adding oxaliplatin to 5FU.^21^ However, debates continue regarding the effect of age on the efficacy of adjuvant therapies [6, 20–22]. To minimize the age bias leading to administering adjuvant chemotherapy, we performed the Cox regression model, adjusted by age, and the result revealed that administering adjuvant chemotherapy did not affect the potentiality for increasing overall or disease-free survival (P = 0.442, 0.997, respectively).

In conclusion, stage III colon cancer patients in our study with only one LN metastasis are a unique group of patients, and differ from those with more advanced stage III disease. We documented that a preoperative CEA level ≤ 5 ng/mL and curative surgery with adequate lymphadenectomy (LN ≧ 12) are favorable prognostic indicators for OS in stage III colon cancer patients who had only one LN (N1a) metastasis. Using postoperative adjuvant chemotherapy in this unique group of patients does not appear to result in different outcomes.

## Supporting Information

S1 Dataset (PDF)
