# Supplementary material for: Overall Survival of Stage III Colon Cancer with Only One Lymph Node Metastasis Is Independently Predicted by Preoperative Carcinoembryonic Antigen Level and Lymph Node Sampling Status
Source: PLoS One. 2015 Sep 18;10(9):e0137053. doi: 10.1371/journal.pone.0137053 (PMC4575069; doi:10.1371/journal.pone.0137053)
Supplement: S1 Dataset — (PDF) [file pone.0137053.s001.pdf]

Sex male=1	Age	Performance	Survival time	Death=1	Disease-free time	Recurren=1	LN number	Grading
1	81	2	44	0	47	1	10	2
1	71	0	20	1	20	0	2	2
1	59	0	21	1	15	1	13	2
1	83	0	30	0	30	0	6	2
1	73	0	22	1	14	1	13	2
1	76	0	25	1	25	0	3	2
2	62	0	103	0	103	0	12	2
2	61	0	102	0	102	0	23	2
2	68	0	99	0	97	0	31	2
2	69	0	99	0	99	0	7	2
2	48	0	97	0	97	0	96	2
1	78	0	18	0	18	1	13	2
1	63	0	99	0	99	0	7	2
2	50	0	29	1	25	1	6	2
2	53	0	12	1	2	1	3	2
1	72	0	61	0	61	0	32	2
1	57	0	97	0	8	1	56	2
1	76	0	19	1	9	1	5	2
1	53	0	73	0	73	0	44	2
1	74	0	48	0	48	0	22	2
1	68	0	93	0	93	0	16	2
2	63	0	96	0	96	0	15	2
2	66	0	7	1	5	1	40	2
1	62	0	95	0	85	1	13	2
2	56	0	95	0	95	0	8	2
2	48	0	26	1	16	1	5	2
1	56	0	32	1	17	1	20	2
2	76	0	94	0	94	0	12	2
1	58	0	92	0	92	0	21	2
2	63	0	94	0	94	0	25	2
2	79	1	22	0	22	0	27	2
2	59	0	93	0	93	0	12	2
2	51	0	92	0	75	1	62	2
1	55	0	87	0	87	0	16	2
1	54	0	56	1	46	1	6	2
1	51	0	20	1	16	1	2	2
1	53	0	58	0	5	1	12	2
2	75	0	64	0	64	0	6	2
2	68	0	50	0	50	0	13	2
2	64	0	18	1	12	1	12	2
1	57	0	79	0	22	1	18	2
1	50	0	36	1	15	1	25	2
1	74	0	31	1	31	0	25	2
1	56	0	87	0	87	0	33	2

2	60	0	13	0	12	1	8	2
1	60	0	85	0	85	0	12	2
1	72	0	18	1	12	1	18	2
1	42	0	84	0	84	0	15	2
2	67	0	86	0	86	0	8	2
2	73	0	49	1	39	1	5	2
2	71	0	0	0	0	0	5	2
2	67	0	38	1	29	1	17	2
1	67	0	85	0	85	0	15	3
1	69	0	74	0	74	0	5	2
1	46	0	45	1	22	1	11	2
1	55	0	54	1	42	1	26	2
2	79	0	82	0	82	0	19	2
1	54	0	83	0	83	0	13	2
1	51	0	81	0	81	0	13	2
2	79	0	38	0	38	0	12	2
2	64	0	85	0	85	0	10	2
1	54	0	85	0	85	0	13	2
2	48	0	69	1	17	1	3	2
1	52	0	85	0	85	0	11	2
1	70	0	85	0	85	0	10	2
1	80	1	2	0	2	0	6	2
1	76	0	85	0	85	0	5	2
2	52	0	86	0	86	0	21	2
1	68	0	6	1	3	1	25	2
1	62	0	78	0	78	0	7	2
1	59	0	78	0	78	0	32	2
1	56	0	76	0	76	0	20	2
1	74	0	12	0	8	1	1	2
1	59	0	82	0	10	1	14	2
1	51	0	47	1	16	1	13	2
1	62	0	19	0	19	0	15	2
1	68	0	10	1	6	1	16	2
2	51	0	81	0	81	0	86	2
1	60	0	78	0	78	0	4	2
1	76	0	38	0	38	0	13	2
2	75	0	40	0	6	1	8	2
1	76	0	49	1	48	1	19	2
1	74	0	52	0	38	1	13	2
2	51	0	64	0	64	0	15	3
2	74	0	75	0	75	0	28	2
2	69	0	74	0	74	0	7	2
2	47	0	74	0	74	0	23	2
2	68	0	66	0	66	0	14	2
2	77	0	38	1	38	0	11	2
2	49	0	54	1	34	1	16	2

1	56	0	75	0	75	0	7	2
2	60	0	66	0	66	0	13	2
2	76	0	39	0	39	0	12	2
2	84	1	20	0	20	0	6	2
2	52	0	10	0	10	0	18	2
1	81	0	30	1	27	1	12	2
1	58	0	4	0	4	0	4	3
2	44	0	1	0	1	0	45	2
1	46	0	12	1	10	1	12	2
2	59	0	11	0	11	0	5	2
1	70	0	1	0	1	0	20	2
1	53	0	68	0	68	0	16	2
1	74	0	72	0	72	0	3	2
1	68	0	21	1	16	1	10	2
1	53	0	65	0	65	0	14	2
2	46	0	68	0	20	1	29	2
2	86	1	18	1	10	1	9	2
1	65	0	49	1	26	1	44	2
1	69	0	68	0	68	0	5	2
2	52	0	11	1	11	1	14	2
2	55	0	39	0	18	1	8	2
1	86	1	22	1	22	1	8	2
1	86	1	6	1	1	1	9	2
2	40	0	10	1	10	0	55	2
1	69	0	67	0	25	1	1	2
1	89	0	66	1	24	1	16	1
2	60	0	59	0	59	0	27	2
2	64	0	36	0	30	1	3	2
2	75	0	36	0	16	1	19	3
1	62	0	66	0	66	0	21	2
1	70	0	38	0	35	1	9	3
2	57	0	2	0	2	0	29	2
2	58	0	23	0	23	0	12	2
1	70	0	65	0	30	1	11	2
2	62	0	65	0	51	1	5	2
1	59	0	63	0	36	1	10	2
2	64	0	62	0	62	0	60	2
2	94	1	2	0	2	0	14	2
1	54	0	62	0	62	0	33	2
1	58	0	24	1	23	1	3	2
2	55	0	17	0	17	0	16	2
2	74	0	63	0	63	0	25	2
1	63	0	9	1	9	1	1	3
1	74	0	64	0	64	0	14	2
2	70	0	55	0	55	0	14	2
1	60	0	58	0	58	0	9	2

1	67	0	63	0	63	0	10	2
1	57	0	36	1	28	1	16	2
2	66	0	36	0	36	0	13	2
2	52	0	61	0	61	0	55	2
1	80	0	61	0	61	0	12	2
2	70	0	56	0	19	1	23	2
1	59	0	60	0	34	1	4	2
1	85	1	34	0	34	0	30	2
1	54	0	57	0	57	0	9	2
1	73	0	61	0	61	0	3	2
1	60	0	16	1	13	1	16	2
2	96	2	3	0	3	0	22	2
1	70	0	33	1	6	1	6	2
2	58	0	43	1	28	1	31	2
2	66	0	1	0	1	0	16	2
1	52	0	58	0	30	1	10	2
2	39	0	60	0	60	0	3	2
1	63	0	3	0	3	0	22	2
2	62	0	58	0	58	0	16	2
1	69	0	55	0	55	0	25	2
1	64	0	57	0	57	1	11	2
2	47	0	55	0	38	0	9	2
1	55	0	31	1	8	1	35	2
1	82	0	57	0	57	0	24	2
2	77	0	50	1	46	1	11	2
2	77	0	15	1	4	1	10	2
1	45	0	32	1	11	1	19	2
1	38	0	19	1	13	1	25	2
2	82	0	56	0	56	0	16	2
2	81	0	57	0	57	0	21	2
1	63	0	54	0	54	0	19	2
2	34	0	56	0	56	0	17	2
1	58	0	57	0	21	1	20	2
1	81	0	19	0	19	0	7	2
1	60	0	56	0	56	0	10	2
2	83	0	55	0	55	1	12	2
2	69	0	55	0	55	0	12	2
2	51	0	52	0	52	0	45	2
2	73	0	49	0	49	0	21	3
1	51	0	49	0	49	0	18	2
1	63	0	50	0	3	1	8	2
1	65	0	14	0	14	0	31	2
2	76	0	51	0	51	0	33	2
1	77	0	22	1	13	1	11	2
1	73	0	52	0	52	0	8	2
1	82	0	51	0	51	0	34	2

1	29	0	25	0	2	1	15	2
1	74	0	24	1	24	0	9	2
2	73	0	44	0	44	0	5	2
1	44	0	28	1	28	0	7	2
1	80	0	43	0	11	1	73	2
2	79	0	50	0	50	0	13	2
1	78	0	3	0	3	0	8	2
2	65	0	45	0	45	0	23	1
2	64	0	49	0	49	0	23	2
1	44	0	48	0	48	0	30	2
1	71	0	23	0	23	0	16	2
1	76	0	38	1	21	1	8	2
2	24	0	13	1	7	1	27	2
1	51	0	49	0	49	0	12	2
2	47	0	47	0	47	0	38	2
2	84	0	48	0	2	1	8	2
2	77	0	3	0	3	0	15	2
1	68	0	48	0	48	0	6	2
1	57	0	21	1	12	1	7	2
1	73	0	48	0	39	1	5	2
2	70	0	45	0	45	0	19	2
2	73	0	17	0	17	0	10	2
1	60	0	41	0	15	1	6	2
2	59	0	43	0	43	0	22	2
1	80	0	4	1	4	0	37	2
2	75	0	46	0	46	0	15	2
1	76	0	10	1	3	1	19	2
2	76	0	13	1	11	1	8	2
1	77	0	22	0	22	0	9	2
2	73	0	44	0	44	0	24	2
1	56	0	44	0	44	0	28	1
1	35	0	33	0	33	0	7	
2	87	2	15	1	15	0	14	2
2	53	0	45	0	45	0	2	2
2	77	0	43	0	43	0	16	2
2	62	0	31	1	12	1	17	2
2	47	0	43	0	43	0	5	2
2	77	0	6	0	0	1	9	2
1	56	0	42	0	42	0	39	1
1	80	0	43	0	43	0	28	2
1	49	0	42	0	42	0	8	2
1	67	0	41	0	41	0	29	2
1	49	0	9	0	9	0	12	2
2	53	0	33	0	31	1	12	2
1	52	0	42	0	42	0	8	2
2	85	1	42	0	42	0	20	2

2	57	0	41	0	41	0	3	2
2	61	0	42	0	42	0	40	2
2	80	0	42	0	42	0	33	2
1	81	0	40	0	40	0	12	2
2	68	0	38	0	38	0	15	2
1	64	0	39	0	39	0	10	2
2	62	0	39	0	39	0	3	2
2	45	0	36	0	36	0	26	2
2	70	0	38	0	38	0	17	2
2	65	0	35	0	35	0	17	1
1	51	0	37	0	37	0	17	2
2	71	0	38	0	38	0	14	2
2	60	0	38	0	38	0	25	2
2	68	0	12	0	11	1	37	2
2	83	0	0	1	0	0	6	2
1	57	0	36	0	36	0	17	2
2	65	0	37	0	37	0	11	2
2	51	0	36	0	36	0	7	2
1	71	0	36	0	36	0	11	2
1	58	0	36	0	36	0	9	2
2	81	0	33	0	33	0	9	2
1	58	0	8	0	8	1	11	2
1	81	0	32	0	32	0	12	2
1	54	0	35	0	35	0	8	2
2	68	0	34	0	34	0	14	2
2	66	0	34	0	34	0	28	2
1	26	0	32	0	32	0	8	2
1	50	0	33	0	33	0	52	2
2	66	0	30	0	30	0	19	2
1	59	0	32	0	32	0	30	2
2	62	0	32	0	32	0	17	2
1	60	0	17	0	9	1	34	2
1	87	1	32	0	32	0	18	2
2	71	0	32	0	32	0	17	2
1	71	0	13	0	13	0	4	2
1	80	0	29	0	29	0	28	2
1	73	0	6	1	6	0	11	2
1	81	0	6	0	6	0	12	2
2	89	1	8	0	8	0	8	3
2	67	0	30	0	30	0	28	2
1	72	0	29	0	29	1	15	2
2	77	0	29	0	2	1	20	2
2	65	0	29	0	5	1	22	2
1	42	0	29	0	29	0	23	1
1	66	0	29	0	29	0	12	2
1	70	0	27	0	27	0	52	2

2	74	0	20	1	15	1	26	2
2	32	0	29	0	29	0	10	2
1	85	0	28	0	28	0	11	2
1	58	0	24	0	24	0	18	2
1	83	0	27	0	27	0	13	2
1	80	0	28	0	28	0	22	2
1	59	0	28	0	10	1	35	2
1	51	0	26	0	26	0	20	2
2	66	0	26	0	26	0	14	2
2	71	0	27	0	22	1	15	2
2	65	0	23	0	4	1	15	2
1	36	0	26	0	26	0	36	2
1	31	0	26	0	26	0	25	2
1	67	0	26	0	7	1	11	2
2	76	0	27	0	27	0	12	2
1	48	0	25	0	25	0	23	2
2	67	0	26	0	26	0	28	2
1	62	0	13	0	13	0	10	2
2	54	0	25	0	25	0	9	2
2	52	0	7	1	7	1	13	1
1	64	0	25	0	25	0	13	2
1	62	0	23	0	23	0	25	2
1	67	0	23	0	23	0	37	2
1	58	0	22	0	22	0	20	3
2	68	0	25	0	17	1	17	2
2	64	0	15	1	2	1	22	2
1	57	0	24	0	24	0	16	2
2	74	0	23	0	23	0	27	2
2	66	0	22	0	22	0	28	2
2	35	0	15	0	15	0	22	2
1	67	0	23	0	7	1	20	2
1	69	0	15	0	15	0	15	2
1	74	0	15	1	14	1	10	2
2	49	0	22	0	22	0	34	2
1	60	0	23	0	10	1	28	2
1	57	0	23	0	23	0	16	2
1	45	0	21	0	21	0	14	2
1	39	0	22	0	14	1	25	2
1	84	0	21	0	4	1	19	2
1	51	0	20	0	20	0	28	2
1	63	0	22	0	5	1	25	2
1	62	0	22	0	7	1	3	2
2	60	0	21	0	21	0	23	2
1	60	0	20	0	20	0	18	2
2	55	0	3	0	3	0	24	2
1	56	0	20	0	20	0	23	2

2	80	0	20	0	20	0	22	2
2	57	0	20	0	7	1	28	2
1	69	0	20	0	20	0	20	2
1	60	0	19	0	19	0	16	2
2	31	0	17	0	17	0	23	2
2	68	0	18	0	18	0	12	2
1	64	0	19	0	17	1	20	2
2	54	0	18	0	6	1	8	2
1	52	0	15	0	6	1	29	2
1	73	0	18	0	18	0	12	2
1	54	0	18	0	10	1	16	2
1	64	0	17	0	12	1	10	2
2	50	0	17	0	17	0	15	2
2	83	1	15	0	15	0	16	2
2	61	0	15	0	15	0	35	2
2	77	0	16	0	16	0	16	2
2	70	0	17	0	17	0	29	2
1	75	0	17	0	17	0	18	2
1	67	0	15	0	2	1	16	2
1	68	0	15	0	15	0	16	2
1	70	0	13	0	6	1	19	2
2	36	0	14	0	14	0	20	2
1	89	1	15	0	1	1	12	2
1	68	0	15	0	15	0	11	2
2	53	0	14	0	14	0	14	2
2	58	0	14	0	14	0	22	2
2	32	0	13	0	13	0	50	2
1	46	0	14	0	14	0	11	2
1	70	0	13	0	5	1	13	2
1	78	0	13	0	13	0	15	2
2	86	1	13	0	11	1	21	2
2	76	0	13	0	13	0	68	2
2	47	0	12	0	4	1	20	2
1	59	0	11	0	11	0	19	2
1	74	0	13	0	13	0	20	2
1	57	0	12	0	12	0	15	2
2	75	0	12	0	12	0	31	2
1	64	0	12	0	10	1	9	2
2	58	0	12	0	8	1	32	2
2	52	0	11	0	11	0	20	2
1	60	0	12	0	12	0	36	2
1	50	0	10	0	10	0	17	2
2	79	0	4	0	4	0	7	2

T1,2=1; T3,4=2	Intra-tumor invasion	CEA	0=no chemo; 1=5FU; 2=oxalip	Tumor site; 1=right; 2=left, 3=Sigmoid
2	0	4.56	1	3
2	1	3.03	1	3
2	1	3.27	1	2
2	1	1.13	0	3
2	1	154.8	2	3
2	1	0.95	1	2
2	1	3.49	1	1
2	1	4.69	1	3
2	1	43.5	1	2
2	1	9.5	1	1
2	1	0.57	1	3
2	1	5.53	1	2
2	1	3.39	1	2
2	0	12.5	1	3
2	1	39.4	2	1
2	0	3.99	1	2
2	1	2.41	1	2
2	1	391.3	1	1
2	1	3.36	0	2
2	1	16.9	1	1
2	0	1.42	1	2
2	1	56.8	1	1
2	1	50.1	0	2
2	0	0.49	0	1
2	1	11.7	1	2
2	1	10.5	2	3
2	1	6.09	1	2
2	1	14.6	1	3
2	1	14.6	0	2
2	0	2.77	0	2
2	1	6.6	1	2
2	1	1.01	2	1
2	1	4.15	1	2
1	0	2.07	1	3
2	1	2.47	1	2
2	1	3.12	0	3
2	1	9.69	0	2
2	1	1.67	1	2
2	1	22.8	0	2
2	1	536.5	1	1
2	1	3.45	1	2
2	1	107.3	1	1
2	1	5.73	1	1
2	0	1.49	1	3

2	1	74	1	3
2	1	2.14	0	3
1	1	3.24	1	2
2	1	1.92	0	2
2	1	398.7	1	1
2	0	7.69	1	2
2	1	139.9	0	3
2	1	5.3	1	2
2	1	4.12	1	1
2	1	4.35	0	1
2	0	3.52	0	2
1	1	2.62	1	3
2	1	2.45	0	1
2	0	2.63	2	3
1	1	3.74	1	2
2	1	6.44	1	3
2	1	1.05	0	2
2	1	3.22	0	2
1	1	2.28	1	3
2	1	2.01	1	3
2	0	1.7	2	2
2	1	3.45	0	2
2	1	1.64	1	1
2	0	12.1	0	1
2	1	90.1	0	1
2	1	6.79	0	2
2	1	2.62	1	2
2	0	2.71	1	3
1	0	6.26	1	1
2	0	1.06	2	2
2	1	5.58	1	2
2	1	14	2	3
1	1	9.43	1	1
2	0	0.32	2	2
2	1	2	1	1
2	1	31.6	1	3
2	1	38715	1	2
2	0	8.8	1	2
2	1	5.85	1	1
2	1	2.6	1	2
2	0	4.65	1	2
2	1	1.88	1	2
1	1	1.31	1	2
2	1	2.26	2	2
2	0	30.9	0	2
2	0	0.51	2	1

2	1	4.02	2	3
2	1	16.4	1	2
2	1	3.27	1	1
2	1	2.11	0	2
2	1	2.61	1	3
1	1	30.3	1	2
2	1	3525.9	0	1
2	0	2.21	0	2
2	0	2.72	1	2
1	1	17.2	1	2
2	1	2.53	0	3
2	0	2.02	0	2
2	0	0.58	1	1
2	1	247.8	1	2
2	1	3.4	0	2
2	0	2.24	0	1
2	1	3.67	1	1
2	0	0.63	1	1
2	1	1.4	1	3
1	1	1116.9	1	3
2	0	5.43	1	1
2	1	5.83	1	3
2	0	283.7	1	3
2	1	0.1	1	2
1	1	0.92	0	3
2	1	0.7	1	1
2	1	2.38	1	3
2	1	3.83	1	1
2	1	0.1	1	2
2	1	0.7	1	2
2	0	1.27	1	2
1	1	2.02	1	3
2	0	1.02	1	2
2	1	11.3	1	3
1	1	1.35	1	2
2	0	0.1	1	2
2	1	1.89	1	2
2	1	2.21	0	1
2	1	9.92	1	1
2	1	25.7	1	3
2	0	1.5	0	3
2	0	3.25	1	1
2	1	3.28	1	2
2	1	2.01	1	3
2	0	3.21	1	2
1	0	3.24	1	1

2	1	4.14	1	1
2	1	0.15	1	2
2	1	1.99	1	2
2	1	0.89	1	1
2	1	2.41	1	1
2	0	14.9	1	1
2	1	0.88	1	3
2	1	1.67	1	2
2	0	0.48	1	1
2	1	0.64	1	1
2	1	7035.8	1	2
2	1	3.77	0	2
2	0	3.13	2	1
2	1	6.22	1	2
2	1	27.7	0	1
2	1	38.3	1	1
2	0	0.24	1	2
1	1	4.87	1	3
2	1	2.14	1	1
2	1	1.1	1	2
2	0	2.41	2	1
2	1	0.98	1	2
2	1	9.47	2	3
2	1	2.69	1	1
2	1	1.76	2	2
2	0	16.2	2	2
2	0	1.07	1	1
2	0	12	1	1
2	1	28.6	2	1
2	0	11.4	1	2
1	1	1.09	1	1
2	1	0.1	2	2
1	0	132.4	1	2
2	1	5.23	1	2
2	1	2.1	1	2
2	0	2.62	1	1
2	0	0.82	1	2
1	1	9.98	2	1
1	1	4.33	1	3
2	0	0.11	2	1
2	1	3.64	1	3
2	0	2.98	1	2
2	0	1.78	1	1
2	1	67.5	2	1
2	0	1.81	1	2
1	1	4.1	1	2

2	1	25.1	1	1
2	0	1.97	1	1
2	1	7.17	1	1
2	1	6.83	1	2
2	1	23.1	1	1
2	0	0.62	1	1
2	1	11.2	1	1
2	1	0.81	2	2
2	1	4.66	2	2
2	1	0.1	2	1
2	1	141.7	1	2
2	1	1	2	3
2	1	1734.8	2	2
2	1	0.71	2	2
2	1	1.09	2	3
2	1	2.32	0	2
2	1	0.53	1	2
2	1	0.71	1	1
2	1	2.2	2	3
2	0	1.31	1	3
1	1	0.1	2	3
1	0	2.45	0	3
2	1	2.43	1	1
2	1	1.92	2	2
2	1	2.55	0	3
2	1	5.92	1	1
1	1	74.7	1	2
2	1	0.89	0	1
2	1	1.08	1	2
2	0	2.75	0	2
1	1	1.05	1	1
1	1	2.8	1	1
2	0	1.23	0	3
2	1	4.4	1	1
2	1	0.75	1	2
2	1	2.31	1	2
1	1	3.72	1	3
2	1	130.7	0	2
2	1	10.1	1	1
2	1	0.97	1	1
2	1	2.04	1	2
2	1	1.11	1	3
2	1	1.12	1	1
2	1	2.42	1	3
2	1	3.68	1	2
2	1	5.71	0	3

2	1	5.9	1	2
2	0	1.19	1	3
2	1	0.68	1	1
2	1	5.56	1	1
2	1	0.48	1	2
2	1	3.96	2	2
2	0	2.54	1	2
2	0	13.8	2	3
2	0	1.66	1	1
2	0	0.97	1	1
2	0	1.86	2	2
2	0	2.08	2	1
2	1	1.68	1	2
2	1	15	1	2
2	0	4.09	0	1
2	0	4.73	1	2
2	0	4.59	1	2
2	0	1.73	1	2
2	0	0.9	1	3
2	0	1.38	1	3
2	1	15	1	1
2	0	8689.2	2	3
2	1	2.86	1	2
2	0	1.74	1	3
2	1	6.17	2	3
2	1	1.92	1	3
2	0	0.47	1	1
2	1	2.72	1	2
2	0	11.3	2	2
2	1	0.13	1	2
2	1	1.76	1	2
2	0	0.73	2	3
1	1	1.58	1	1
2	0	7.29	1	3
2	0	3.17	0	1
1	0	2.53	1	3
2	0	0.54	0	1
1	1	2.21	0	1
2	0	2.72	1	3
2	1	24.5	1	1
2	1	5.43	1	2
2	1	98.7	1	3
2	1	355.1	1	1
2	0	1.19	1	1
1	1	2.62	0	2
2	0	2.35	0	3

2	1	49.7	1	2
2	1	4.36	1	1
2	1	1.28	1	2
1	0	9.75	2	1
2	0	3.51	1	3
1	1	3.96	1	3
1	0	1.88	2	1
1	1	3.92	1	1
2	1	0.49	0	2
2	1	6.96	1	2
2	1	2.8	1	2
2	0	29.6	2	3
2	0	1.59	2	1
2	1	4.49	1	1
2	1	4.17	1	1
2	1	2.67	1	1
2	1	1.76	1	2
1	0	2.86	1	3
1	1	8.17	1	2
2	0	1339	1	3
1	0	1.23	2	2
2	0	0.97	0	2
1	1	1.89	2	3
2	1	0.44	2	2
2	1	22.1	2	1
2	1	17.7	1	2
2	1	0.1	2	2
2	1	1.24	1	1
2	0	1.9	1	1
1	0	1.46	2	1
1	0	16.5	2	1
2	0	5.8	1	2
2	1	1.5	2	3
2	1	4.9	2	3
2	1	0.3	2	2
2	1	3.44	2	1
2	1	0.1	1	3
2	1	2.24	1	2
2	1	2.45	1	1
2	0	0.43	0	1
2	0	6.08	1	3
2	1	30	2	2
2	1	1.91	2	3
2	1	2.89	2	1
1	0	1.94	2	3
1	1	1.07	0	1

2	1	52.2	2	1
1	1	1.35	1	3
2	0	3.29	2	2
2	1	2.16	0	2
1	1	1.11	2	3
2	0	0.48	2	2
1	1	3.98	1	1
2	1	10.9	1	3
2	1	4.41	2	2
2	0	1.69	1	1
1	1	0.43	2	3
1	1	7.6	2	3
2	1	0.9	2	2
2	0	0.32	0	2
2	0	3.47	0	2
2	1	28.7	1	1
2	1	1.17	2	2
2	1	0.51	1	2
2	1	1.73	2	1
2	0	4.87	1	2
2	1	275.6	1	2
2	1	0.43	2	2
2	1	56.1	1	2
2	0	1.09	1	2
1	0	0.47	2	2
2	1	1.7	2	2
1	1	1.44	1	1
2	1	1.51	1	1
1	1	7.97	2	3
2	1	143.6	1	2
2	1	0.93	1	2
2	1	2.7	1	1
1	1	23.2	1	1
2	1	3.42	0	3
2	1	0.84	1	2
2	1	1.18	2	3
2	0	9.86	1	2
2	0	3.42	2	1
2	1	0.91	2	3
2	0	9.29	1	1
2	0	1.3	0	3
2	0	2.27	1	1
2	0	0.62	1	3
